# Sustainable Practices and Microbial Quality of Cattle Offal in Slaughterhouses

**DOI:** 10.3390/vetsci12020153

**Published:** 2025-02-11

**Authors:** Ana Raquel Cândido, Kamila Soares, Márcio Moura-Alves, Cristina Saraiva, Alexandra Esteves

**Affiliations:** 1 PEC Nordeste, Zona Industrial II, 4564-909 Penafiel, Portugal; qualidade@pecnordeste.pt; 2 Veterinary and Animal Research Center (CECAV), University of Trás-os-Montes e Alto Douro (UTAD), 5000-801 Vila Real, Portugal; kamilas@utad.pt (K.S.); mmalves@utad.pt (M.M.-A.); crisarai@utad.pt (C.S.); 3 Al4AnimalS Associated Laboratory for Animal and Veterinary Science, 5000-801 Vila Real, Portugal; 4 Department of Veterinary Science, School of Agrarian and Veterinary Science (ECAV), University of Trás-os-Montes e Alto Douro (UTAD), 5000-801 Vila Real, Portugal

**Keywords:** food safety, offal, tongue, liver, cattle slaughterhouse

## Abstract

As global income levels and population increase, meat consumption and production are rising, raising concerns about climate change, animal welfare, and environmental sustainability. Offal offers a sustainable, nutrient-rich, and economical alternative to muscle meat, providing proteins, vitamins, and minerals. This study was triggered by the scarcity of information on the microbiological quality of edible bovine offal, which is often associated with poor hygienic conditions during handling and processing at slaughterhouse. This study assessed the superficial microbiota present on cattle liver and tongue in a vertical slaughterhouse across various stages of the procurement process and on days with different slaughter volumes, with three sampling points: immediately after post-mortem inspection (Point 1); after the arrival of the viscera in the preparation room (Point 2); and in the refrigeration chamber at 3 °C (Point 3). Samples were analysed according to ISO methods for enumeration of mesophilic aerobic bacteria, *Enterobacteriaceae*, generic *Escherichia coli*, presumptive *Escherichia coli* O157, and *Staphylococcus aureus* and detection of presumptive *Escherichia coli* O157, *Staphylococcus aureus*, and *Salmonella* spp. Microbiological counts were within the acceptability values stipulated in the amendment of Regulation EC 1441/2007 to Regulation EC 2073/2005 for cattle carcasses. In general, both viscera showed higher microbial counts on days with higher slaughter volumes, with the exception of *Enterobacteriaceae* and *Staphylococcus* counts on the tongue. There was also an increase in microorganism levels across the offal processing stages. Out of 144 samples, 20.83% were contaminated with presumptive *Escherichia coli* O157, 3.47% *Salmonella* spp., and 25% *S. aureus*. Therefore, although 20.83% of the samples tested were presumptive for *E. coli* O157, it is important to note that a considerable number of these presumptive positives may in fact have been negative after confirmation by molecular methods. However, the presence of presumptive *E. coli* O157 at high levels is one reason that the prevention of offal contamination requires more stringent measures. Preventing offal contamination requires stricter measures during slaughter procedures to ensure safety, reduce food losses, and enhance sustainability in meat production.

## Simple Summary

The goal of this study was evaluation of the microbiological quality of bovine offal (liver and tongue) in a vertical slaughterhouse, focusing on detection of pathogens and contamination levels during processing under different slaughter volumes. Out of 144 samples, 20.83% tested positive for presumptive *Escherichia coli* O157, 3.47% for *Salmonella* spp., and 25% for *Staphylococcus aureus*. While microbial counts were generally within acceptable EU regulatory limits, higher slaughter volumes were correlated with increased contamination. There was also an increase in microorganism levels across the offal processing stages. Preventing offal contamination requires stricter measures during slaughter procedures to ensure safety and reduce food losses to enhance sustainability in meat production.

## 1. Introduction

Due to population growth, the global demand for food is on the rise, demanding an increase in food production and supply coupled with efforts to reduce or eliminate wastage [1]. Meat’s significance stems from its organoleptic qualities and high nutritional value [2]. It is considered an important source of B-complex vitamins and minerals such as iron (Fe) and zinc (Zn), as well as protein, fat, and energy [3,4] Meat is one of the most important sources of human protein requirements [5]. Offal is a dense, rich, and economical source of essential nutrients, such as proteins, vitamins, and minerals, for human consumption [6], and is considered a good alternative to the consumption of muscle meat. In addition, population growth is increasing global consumption of meat and meat products [7]. Consequently, in response to this demand, meat production worldwide is steadily increasing [8].

The increasing pressure from climate change, animal welfare issues, and environmental sustainability regarding the impact of animal slaughter on meat production has become a priority [9,10]. Animal production is widely recognised for its significant environmental impact, primarily through greenhouse gas emissions, intensive land use, and high water consumption. In Europe, livestock production systems account for 73% to 80% of the agricultural sector’s environmental impacts, affecting water quality, biodiversity, air quality, soil acidification, and global warming. To achieve meaningful environmental improvements, it is essential not only to address livestock production but also to consider dietary choices and food waste in Europe [9]. Contemporary consumers are increasingly attentive to the environmental footprint associated with food production [11]. Consequently, there is a pressing need for rational and balanced management of meat and its derivatives throughout the entire food supply chain to promote sustainable production and reduce food losses [8].

Animal slaughter entails significant economic costs and environmental repercussions [12,13], resulting in the production of meat, edible offal, and by-products not used for human consumption. The cattle industry generates numerous by-products during the harvesting process. These by-products are categorised into edible offal, which includes organ meats, and inedible offal, comprising blood tissue, fat, hides, hooves, horns, bones, and lungs [14].

This presents an opportunity for the industry to develop value-added products and alternative markets for low-value by-products and products, increasing the profitability of slaughterhouses processing operations [14,15].

For various reasons, the consumption of offal has recently received significant attention worldwide [16]. The world faces a huge problem of food insecurity and climate change, which results in malnutrition; therefore, the viscera from slaughtered animals must be used as food to combat malnutrition [17]. However, a challenge lies in beef offal consumption, due to a significant association between food neophobia and food disgust sensitivity, with neophobia indirectly affecting the intention to consume offal, mediated by disgust sensitivity. Sociodemographic variables such as age, gender, and income influence both neophobia and disgust sensitivity. These findings underscore the importance of strategies to increase offal consumption, suggesting interventions aimed at modifying entrenched behaviour patterns to promote broader acceptance of these foods, considering consumers’ capabilities, opportunities, and motivations [18].

Despite slight variations in offal consumption in Portugal, there was an increase from 113.4 kg in 2020 to 119.6 kg in 2023. In 2023, offal consumption represented 5.89% of the total meat and offal consumption [19].

Scare information about the microbiological characteristics of edible bovine offal, and the generally accepted fact that due to readily available nutrients and poor hygienic conditions during handling, collection, and processing, offal can possess poor microbial quality, constituted the trigger for the execution of this study.

Offal can become contaminated by microorganisms during the slaughtering process, particularly during evisceration, as poor hygiene practices facilitate the spread of microbial agents, significantly reducing their shelf life. The contact between red and white viscera is cited as an important cause of contamination [20]. The skinning operation is also considered critical, with contamination potentially occurring through various means: the initial incision, skin inversion, successive handling of the skin and meat by the operator without intermittent handwashing, and contact between the meat and equipment, walls, or floors [21]. Among the pathogens described as a result of these cross-contaminations are *Salmonella*, *Clostridium perfringens*, *Escherichia coli, Staphylococcus aureus*, and *Bacillus cereus* [22]. An EFSA report from 2023 revealed that of 2963 carcass cattle samples collected by food business operators (FBOs) from Portugal, 8 (0.27%) were positive for *Salmonella* [23].

*E. coli* O157 is a primary serotype of the strains that produce toxins known as verotoxins or Shiga-like toxins that cause severe illnesses, particularly in certain vulnerable groups, including children, the elderly, and immunocompromised individuals [24,25,26].

The main goal of this study was to assess the superficial microbiota present on the cattle liver and tongue, considered offal with high economic value, in a vertical slaughterhouse across various stages of the procurement process. Our focus was on identifying potential sources of contamination or procedural factors contributing to this offal contamination. The influence of slaughter volume on the microbiological characteristics of these cattle offal types was also evaluated. The generated information can be used to outline strategies aimed at improving microbiological characteristics in offal production, leading to its extended shelf life and contributing to a reduction in food losses.

## 2. Materials and Methods

### 2.1. Slaughterhouse Layout

The study was conducted in a vertically arranged slaughterhouse located in northern Portugal. A “vertical slaughterhouse” is an architectural and operational concept designed to offer a compact solution for the slaughter and processing of animals in a verticalised environment. This type of structure aims to enhance efficiency, hygiene, and sustainability in meat processing by organizing the processes across different levels of a building. The layout of a vertical slaughterhouse enables a clear separation between clean areas (final products) and dirty areas (e.g., the slaughter line), effectively reducing the risk of contamination [27].

**Figure 1:**
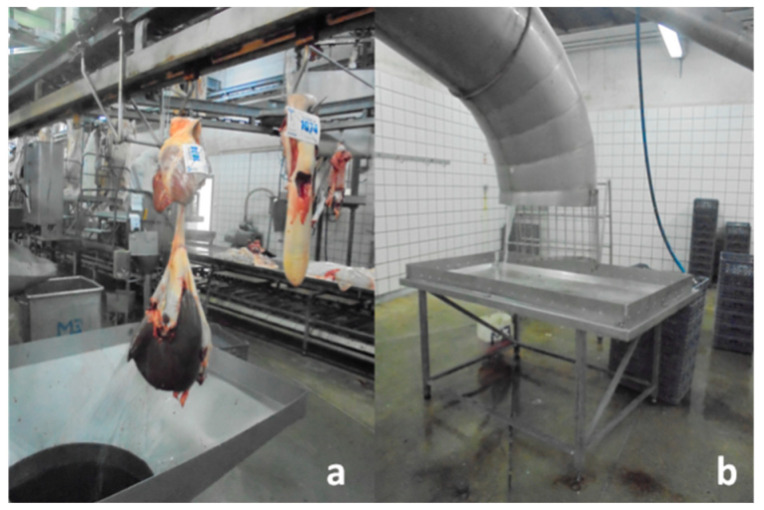
(**a**) Slaughter line (upper floor), viscera transfer hatches; (**b**) Viscera preparation room (lower floor), area for trimming the transferred viscera.

On the upper floor, the slaughter line is located, where animals are received, slaughtered, and carcasses are obtained. Subsequently, skins, foot ends, viscera, carcasses, and other by-products are processed as they move “down” through the building’s levels via a system of chutes, often assisted by gravity (Figure 1).

### 2.2. Sample Collection

**Figure 2:**
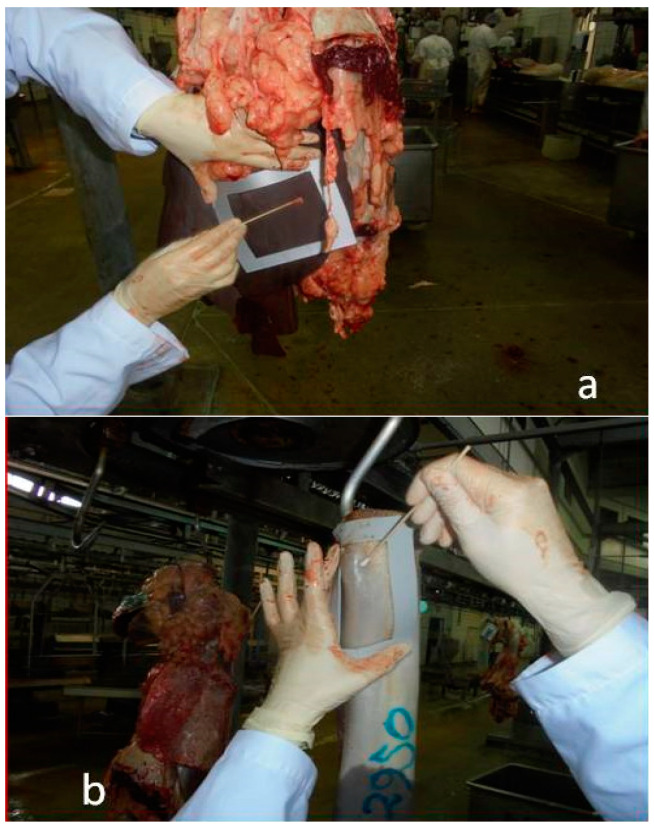
Preliminary sample collection procedure. (**a**) Liver surface sample and (**b**) Tongue surface sample.

During winter, samples from the surface of the liver and tongue (Figure 2) of 24 animals were collected over 6 days, distributed across 3 days with high slaughter volumes (more than 200 animals) and 3 days with low slaughter volumes (fewer than 50 animals), identified as H and L, respectively.

For each viscus, swabs were taken at three different stages of preparation: immediately after post-mortem inspection (Point 1); after the arrival of the viscus in the preparation room (Point 2); and in the refrigeration chamber when it reached 3 °C (Point 3), totalling 144 swabs. The time elapsed between the first and last sampling points on the same viscus was approximately 4 h.

The viscera chosen for the study were from animals with slaughter order numbers roughly half of the total number of animals slaughtered on that day. This approach was taken to reduce the potential impact of the slaughter order on the study results.

Each sample was collected using a sterile swab (VWR, Leuven, Belgium) moistened in tubes with a 10 mL solution of tryptone salt (Himedia, Mumbai, India) for microbial enumeration. For pathogen detection, we used three sterile bags containing a dry, biocide-free cellulose sponge (VWR, Leuven, Belgium) for each microorganism.

The swabs or sponge were then rubbed over a 25 cm^2^ surface of the organ, which was delimited by a previously sterilised gauge. Each swab or sponge was rubbed longitudinally over the delimited area for 20 s.

The collected samples were promptly transported to the laboratory within a maximum of two hours, properly identified, and refrigerated. In the laboratory, the samples were refrigerated at a temperature of 4 °C for a maximum period of 24 h after the first sampling, following the protocol outlined in International Organization for Standardization (ISO) 17604/2015 [28].

### 2.3. Microbiological Analysis

The samples were analysed for detection for presumptive *E. coli* O157, *Staphylococcus aureus,* and *Salmonella* spp. and enumeration of total mesophilic aerobic bacteria (TMB), *Enterobacteriaceae* (ENT), generic *E. coli*, presumptive *E. coli* O157, and *S. aureus*.

#### 2.3.1. Pathogen Detection

The detection of *Salmonella* was conducted according to ISO 6579:2017 standards [29] with modifications. Sponge samples were suspended in 25 mL of buffered peptone water medium (VWR, Leuven, Belgium). The samples were then incubated at 37 °C for 24 h. Subsequently, selective enrichments were performed using Rappaport–Vassiliadis broth (RV) (LIOFILCHEM, Roseto degli Abruzzi, Italy), which were incubated at 41.5 °C for 24 h, and Müller–Kauffmann tetrathionate–novobiocin (MKT) (LIOFILCHEM, Roseto degli Abruzzi, Italy), which were incubated at 37 °C for 24 h. After incubation, the contents of the MKT and RV tubes were spread onto Hektoen enteric agar (HEKT) (VWR, Leuven, Belgium) and CHROMagar™ Salmonella plus (CHROMagar, Paris, France) and incubated at 37 °C for 18–24 h. After detection of typical colonies, five colonies were selected from each sample, and subcultured onto nutrient agar (NA) (HIMEDIA, Mumbai, India) and incubated at 37 °C for 24 h. Subsequently, biochemical tests were performed to assess sugar fermentation on triple sugar iron agar (TSI), urease activity, lysine decarboxylation, and indole production. The results of these biochemical tests, along with the observation of typical characteristics on CHROMagar medium, were used confirm the presence of *Salmonella*. For control analysis, a reference strain, *S. Typhimurium* (C6000, OXOID, batch 603220), was used. The detection of presumptive *E. coli* O157 was performed by suspending the sponge sample in 25 mL of modified tryptone soya broth with novobiocin (mTSB base) (BIO-KAR, Beauvais, France) and incubating at 41.5 °C for 18–24 h. Selective enrichment was prepared using immunomagnetic beads (Dynabeads, Thermo Fisher Scientific, Waltham, MA, USA) to isolate *E. coli* O157. After the separation process, the enriched samples were spread onto MacConkey agar with sorbitol (CT-SMAC) (HIMEDIA, Mumbai, India) and incubated at 37 °C for 18–24 h, according to ISO 16654:2001 [30] standards, and CHROMagar™ O157 (CHROMagar, Paris, France) and incubated at 37 °C for 24 h. Five typical colonies in CT-SMAC were subcultured onto NA, and biochemical tests, such as the indole test, were performed. The results of these biochemical tests, along with the observation of typical characteristics on CHROMagar medium, were used to confirm the presence of *E. coli* O157. For control analysis, the reference strain was used *E. coli* O157:H7 (VT(n)—C1967, OXOID, batch 607914, UK).

The detection of *S. aureus* was performed by suspending the sponge sample in 25 mL of Chapman broth (Section S1 in Supplementary Materials) and incubating it at 37 °C for 24 to 48 h. After the incubation period, the samples were spread onto Baird-Parker agar (BPA) medium with egg yolk–tellurite emulsion 20% and rabbit plasma fibrinogen supplement II (RPF) (BIOLIFE, Milano, Italy) and incubated at 37 °C for 24 h. If necessary, incubation was extended beyond 24 h, according to ISO 6888-2:1999 [31]. Five typical colonies in BPA/RPF were also subcultured onto CHROMagar™ Staph aureus medium (CHROMagar, Paris, France) and incubated at 37 °C for 18–24 h. The results of the BP/RPF and CHROMagar medium were used to confirm the presence of *S. aureus*. For control analysis, the reference strain used was *S. aureus* (ATCC^®^ 6538, NCTC 10788, TCS Biosciences Ltd., batch 46/37, UK).

#### 2.3.2. Enumeration of Bacteria

The samples were collected using a sterile swab into tubes with a 10 mL solution of tryptone salt and then homogenised for 60 s using a vortex. The homogenates were then serially diluted and 1 or 0.1 mL portions of the diluted suspensions were pour-plated by incorporation or surface-plated on non-selective and selective agar plates. To quantify different groups of bacteria, the following media and conditions were used: plate count agar (PCA) (VWR, Leuven, Belgium) at 30 °C for 72 h for the enumeration of TMB, according to ISO 4833-1:2013 [32], and violet red bile glucose agar (VRBG) (VWR, Leuven, Belgium) at 37 °C for 24 h for ENT (ISO 21528–2:2017 [33]). After observing these typical characteristics, confirmation was performed using the oxidase test, where a negative result supports the identification of ENT. Tryptone bile X-glucuronide agar (TBX) (LIOFILCHEM, Roseto degli Abruzzi, Italy) at 44 °C for 24 h was used for generic *E. coli*. MacConkey agar with sorbitol (CT-SMAC) (HIMEDIA, Mumbai, India) at 37 °C for 18–24 h was used for presumptive *E. coli* O157. Five colonies were subcultured in NA (HIMEDIA, Mumbai, India), incubated for 37 °C for 24 h, then subcultured in CHROMagar™ O157 (CHROMagar, Paris, France), and indole tests were performed for confirmation. Baird-Parker agar (BPA) with egg yolk–tellurite emulsion 20% and rabbit plasma fibrinogen supplement II (RPF) (BIOLIFE, Milan, Italy) at 37 °C for 24–48 h was used for coagulase-positive staphylococci. Five colonies were also subcultured onto CHROMagar™ Staph aureus medium (CHROMagar, Paris, France) and incubated at 37 °C for 18–24 h for confirmation.

All results are expressed as log CFU/cm^2^.

### 2.4. Statistical Analysis

Statistical analysis of the obtained results was performed using IBM SPSS^®^ Statistics version 7 (STATISTICS 7). The distribution and data normality were evaluated through the Shapiro–Wilk test. The enumeration results are expressed as log CFU/cm^2^. The detection frequencies of presumptive *E. coli* O157, *Salmonella*, and *S. aureus* were analysed by the ratio of positive results and the total number of samples used (number of positive samples × 100/total number of samples).

To evaluate the behaviour of the measurement variable supposedly influenced by a factor or effect with more than two groups, one-way analysis of variance (ANOVA) was used, provided that the assumptions of normality within groups and homogeneity of variances between these groups were met for the numerical characteristic under study. The significance level was set at *p* < 0.05, significant, (*); *p* < 0.01, very significant, (**), and *p* < 0.001, highly significant (***).

The Tukey test was used to establish the difference between the effects of the variances.

## 3. Results and Discussion

### 3.1. Pathogen Detection

Out of 144 offal samples performed, 30 (20.83%) were contaminated by presumptive *E. coli* O157, 5 (3.37%) by *Salmonella*, and 36 (25%) by *S. aureus*.

In 2023, 4128 units of fresh and unprocessed bovine meat were tested by pathogens as per Member States (MSs). Most pathogen specimens sampled at the manufacturing level were taken from slaughterhouses (72.4%, N = 1860), and 6.5% tested positive for STEC [23]. Regarding the prevalence of *Salmonella* in bovine carcasses, Portugal reported 8 positives (0.27%) out of 2963 samples collected by the food business operator (FBOp) [23]. Five MSs—Croatia, Germany, Greece, Italy, and Spain—reported data on *S. aureus* in animals (N = 8913) with 10.8% of the animal samples testing positive, and in food matrices (N = 6700), with 5.4% testing positive [23].

The majority of authors associate the presence of *E. coli* O157 in slaughterhouses with the slaughter of cattle carriers of this microorganism. In an Ethiopian slaughterhouse, the prevalence of *E. coli* O157 in cattle carcass surface swabs was 2.7% (4/150) [34]. Matthews et al. [35,36] observed variability in *E. coli* O157 prevalence on Scottish cattle farms due to environmental factors, animal movement, and carriage levels. Naylor, et al. [37] indicated that *E. coli* O157 colonisation occurs specifically in the distal colon, near the recto–anal junction. Furthermore, a subset of cattle, referred as “super shedders”, excrete high levels of *E. coli* O157:H7 (>10^4^ CFU/g of faeces).

As previously mentioned, slaughtered animals are often asymptomatic carriers of pathogens, and cross-contamination results from inadequate handling practices on the slaughter line, whether from poorly conducted skinning and/or evisceration processes, coupled with deficient hygienic practices during the preparation of carcasses and offal. The concern over the contamination of bovine carcasses by pathogens such as *Salmonella* and pathogenic *E. coli* has led to the recommendation to avoid palpation and incision during post-mortem inspection to reduce the spread of these high-priority biological hazards. Regulation (EU) 2019/627 [38], focuses on mitigating cross-contamination during meat inspections, since it reduces all inspection procedures related to cross-contamination to the essential.

The Regulation EC 1441/2007 amendment of Regulation EC 2073/2005 regarding microbiological criteria for cattle carcass surfaces and Microbiological Standards for Foodstuffs established by the G.C.C Standardization Organization (GSO 1016/2015) [39] for raw edible offal, including liver, kidney, and muscle stomach, require the total absence of detectable *Salmonella.* In the present study, the presence of *Salmonella* was detected in viscus swabs, though with low prevalence.

The observed prevalence of *S. aureus* at 25% in offal swabs, with 36 positive cases, is often linked to handling practices, in addition to the presence of asymptomatic *S. aureus* carriers. As was demonstrated in a study conducted by de Moura, et al. [40], in five public slaughterhouses in Brazil, from a total of 150 swab samples collected, high levels of *S. aureus* contamination were detected on the hands of 50 handlers (90%), 50 utensils and equipment (88%), and 50 cattle carcasses (84%), which indicated the existence of cross-contamination. These results underscore the importance of stringent hygiene practices during handling, indicating that inadequate procedures can significantly contribute to contamination and elevate food safety risks.

The presence of pathogens in swabs of liver and tongue samples is summarised in Table 1, stratified by slaughter volume (high, H; low, L) and sampling points (1, 2, and 3) along the offal preparation line.

In the present study, tongue samples were most frequently contaminated by presumptive *E. coli* O157, *Salmonella*, and *S. aureus*.

Based the type of viscera, presumptive *E. coli* O157 was detected in 19.44% of liver samples (14 positives) and 22.22% of tongue samples (16 positives). *Salmonella* was detected in 1.39% of liver samples (1 positive) and 5.55% of tongue samples (4 positives), while *S. aureus* was found in 19.44% of liver samples (14 positives) and 30.55% of tongue samples (22 positives).

These results may be attributed to the fact that the tongue, being an organ of the gastrointestinal system, can come into contact with regurgitated material from the gastrointestinal tract [41]. Additionally, the tongue remains in contact with the environment even before the animal is slaughtered. These findings support the notion that variables related to transportation and the environment can influence tissue contamination, such as the tongue, by these bacteria, underscoring the importance of proper management practices to mitigate contamination risks in animals destined for slaughter [42,43]. The practice of withholding feed before the transport of cattle is common to reduce soiling during transportation and ensure the cleanliness of the skin and wool at the time of slaughter. However, this represents only a portion of the period without feeding before slaughter [44]. A study examined the impacts of this period, highlighting that fasting reduces faecal contamination and facilitates a more hygienic slaughtering process. However, if fasting is prolonged, it may increase the risk of unwanted bacterial growth. It must be considered that despite the mentioned advantages of the fasting period, animals not slaughtered within 12 h of their arrival at the slaughterhouse must be provided with feed according to Regulation 1099/2009 [45].

Analysing slaughterhouse volume days, it was possible to observe that on higher-slaughter-volume days (H), the occurrence rate of *E. coli* O157 and *S. aureus* was higher in the liver and the tongue. The results observed can be attributed to the fact that on days with higher slaughter volumes, there is a considerable increase in workload, which may contribute to heightened worker fatigue. This in turn could affect attention and adherence to proper hygiene practices. It is well established that the implementation of good hygiene practices and the enforcement of food safety standards in slaughterhouses are essential for ensuring meat safety [46].

The occurrence of *Salmonella* was comparatively lower and only found in samples from days of low (L) slaughter volume. This result may suggest that the presence of *Salmonella* in viscera is unlikely to result from contamination during preparation, which is typically more likely to occur on days with higher slaughter volumes, contrary to our expectations. The *Salmonella* occurrence observed seems to be intricately associated with the slaughtered animal rather than external factors. This interpretation is reinforced by the fact that despite the higher occurrence of *Salmonella* in the tongue samples (5.55%) compared to the liver (1.39%), all animals presenting *Salmonella* in the liver also present *Salmonella* in the tongue.

Regarding the sampling point, immediately after post-mortem inspection (Point 1) the samples exhibited absence of *Salmonella* and a low prevalence of presumptive *E. coli* O157 and *S. aureus* compared to subsequently analysed points. The only exception was noted on the occurrence rate of presumptive *E. coli* O157 in the tongue, with a higher prevalence value at the first point of the offal preparation line, which could be due to the differences in contamination depending on the surface sampled. The presence of pathogenic microorganisms at the first sampling point (Point 1), immediately after post-mortem inspection, may be associated with the fact that *Salmonella* spp., *Campylobacter jejuni/coli, Yersinia enterocolitica,* and verocytotoxin-producing *Escherichia coli* (VTEC) can be present in the gastrointestinal tracts of meat-producing animals, making slaughterhouses, particularly surfaces (hands and equipment), important vehicles for carcass contamination [46].

In a study conducted by Komodromos, et al. [47], from swab samples collected from the carcass surfaces of 106 cattle in Greece immediately after post-mortem inspection, there was *S. aureus* prevalence of 5.70% (6 positive cases). In the present study, all offal samples at Point 1 (after post-mortem inspection) were absent of *Salmonella*.

Microbial contamination tends to accelerate throughout the slaughter process due to the use of contaminated equipment, lines, gloves, and aprons, among others [48]. In agreement with that study, in the present study, in the offal preparation room (Point 2), there was an increase in the number of contaminated viscera, with presumptive *E. coli* O157 observed in 20.83%, *Salmonella* in 4.16%, and *S. aureus* in 29.17% of liver samples. On tongue samples, we also observed an increase in contaminated samples for *Salmonella* with 16.66% and *S. aureus* with 29.16% contaminated samples. The reduction in samples positive for *E. coli* O157 on the tongue could be explained by the locations in which sampling is carried out and differences in microbial load. The obtained results agree with the occurrence of cross-contamination throughout the offal preparation process. The application of good manufacturing and hygiene practices during the slaughter process has an impact on the reduction in food-borne hazards associated with carcasses and viscera [49].

The increase in pathogen-contaminated samples at Point 2 can be attributed to the type of “downward march” slaughterhouse (vertical slaughterhouse) where the work was carried out (Figure 1). In these slaughterhouses, there are piping systems used to transfer viscera from the upper floor (slaughter line), where they are inspected and approved, to the red offal processing and preparation room on the lower floor. All viscera from the slaughtered animals pass through these ducts, coming into contact with the duct walls, potentially facilitating cross-contamination. A hygiene and disinfection plan for these pipes is essential and must be strictly implemented to ensure to reduce viscus cross-contamination.

In the refrigeration chamber (Point 3), there was no change in the number of tongue samples testing positive for presumptive *E. coli* O157, while *S. aureus*-positive samples increased by 25%. Conversely, liver samples showed a decrease in positive cases, with *E. coli* O157 declining by 4.14% and *S. aureus* by 12.5%.

The notable increase in *S. aureus* presence on the tongue between points 2 and 3 may be linked to handling practices during the viscus preparation process. Proper food handling practices are essential to prevent the presence of *S. aureus* in food products, including raw meat [50].

### 3.2. Microbiological Counts

In the absence of specific microbiological criteria for evaluating the microbiological characteristics of viscera, the “Hygiene Criteria for Processes” established for meat and meat products regarding the cattle carcass was used [51]. These criteria state that the average TMB content on cattle carcass surfaces must not exceed the maximum allowable limit of 5.0 log CFU/cm^2^. Similar criteria are outlined in GSO 1016/2015 [39] for raw edible offal, such as liver, kidney, and muscular stomach, where two out of five samples may fall within the range of 5 log CFU/g to 6 log CFU/g, but no sample may exceed 6 log CFU/g. In the present study, no sample exhibited TMB levels considered excessive.

Concerning *Enterobacteriaceae*, a small number of samples exhibited a slightly higher content than the maximum limit prescribed in the Regulation EC 1441/2007 amendment of Regulation EC 2073/2005 for cattle carcass surfaces (2.5 log CFU/cm^2^) [51] Section S2 in Supplementary Materials. It is important to consider that all the reference values used are referents to analyses carried out using a destructive method, usually linked to a higher microbial content compared to the surface swab method used in this study.

In the Moura et al. (2015) study referred to above, there were average ENT counts of 0.669 CFU/cm^2^ detected on 6 out of 50 handlers tested, 1.088 log CFU/cm^2^ on utensils and equipment on 12 out of 50 tested, and 0.532 log CFU/cm^2^ on bovine carcasses in 14 out of 50 tested. These findings underscore the importance of strict hygiene practices during handling, demonstrating that inadequate procedures can significantly contribute to contamination.

A highly significant positive correlation (*p* < 0.001) when the counts of different microbiological populations were correlated Section S3 in Supplementary Materialswas observed, indicating shared sources of contamination and growth conditions. The abovementioned did not occur when *S. aureus* counts were correlated with the other quantified populations. A positive but not significant correlation was observed (*p* > 0.05). This result could be related to the particularity that the primary source of *S. aureus* is the handling by carriers [52,53].

Table 2 presents mean values (±SD) of the counts on liver and tongue samples.

Tongue samples showed slightly higher counts, which were not statistically significant (*p* > 0.05) for any bacterial indicators. The systematic occurrence of higher microbial levels in the tongue samples may be explained by the fact that it is an organ that comes into contact with the environment, thus naturally harbouring its microbiota during the animal’s lifetime and eventually in gastrointestinal content.

Note that the number of counts of *S. aureus* observed could be considered acceptable, considering that enterotoxin production requires a level of *S. aureus* greater than 10^5^. This is the reason that the presence of *S. aureus* at this level in milk and milk products in EU Regulation [51] justifies the need to search for staphylococcal enterotoxins.

However, the high prevalence of *S. aureus* (25%) in the analysed samples alerts us to the need to improve handlers’ practices.

In Table 3a,b and Table 4a,b, the average values (±SD) of the counts performed on the liver and tongue samples are presented according to the slaughter volume and sampling point, respectively.

Both organs showed higher microbial counts on days of higher slaughter volume, with the difference being statistically significant (*p* ≤ 0.01) for TMB, generic *E. coli*, and presumptive *E. coli* O157. Casagrande, et al. [54] presented similar results, indicating that days with higher slaughter volume are associated with higher levels of meat contamination.

On tongue samples, TMB and ENT presented significant differences (*p* < 0.001 and *p* < 0.01, respectively) in microorganism levels when sampling points 1 and 2 were compared, which can be associated, as explained above, with contamination of the tongue during its carriage and preparation in the viscus preparation room (Point 2). However, between sampling points 2 and 3, no significant changes were observed.

In the case of presumptive *E. coli* O157, a gradual increase was observed throughout the sample points analysed, being statistically significant at Point 3.

The average levels of contamination by TMB, ENT, generic *E. coli*, and presumptive *E. coli* O157 tended to increase throughout the preparation process of both liver and tongue, with a significant rise. Immediately after post-mortem inspection (Point 1), the microbial counts observed in both organs are attributable to cross-contamination arising from procedures involved in carcass and organ preparation, as well as frequent handling and inspection procedures, which may include palpation and incision of organs as stipulated by Regulation (EC) 627/2019 [38].

The presence of these microorganisms in the viscera after evisceration at Point 1 can primarily be explained by deficiencies in the preparation of carcasses and viscera at the slaughter line. Specifically, generic *E. coli* and presumptive *E. coli* O157 are part of the natural microbiota found in the intestines of mammals. Faecal contamination is associated with the evisceration process, either through direct exposure to gastrointestinal contents or via cross-contamination from contact with contaminated surfaces, personnel, or equipment. Preventing contact between red and white viscera is imperative to minimize the risk of contamination by these pathogens [35].

Surprisingly, no significant changes were observed in *S. aureus* counts across the different sampling points, unlike the study of Desmarchelier, et al. [55], which reported increased counts of coagulase-positive *Staphylococcus* in cattle carcasses between evisceration procedures and refrigeration.

The statistically significant increase in TMB, ENT, generic *E. coli*, and presumptive *E. coli* O157 from Point 1 to Point 2 (viscus preparation room) can be explained by the added contamination resulting from the contact of the viscera with the surfaces of the bite system used to transport the organs from the slaughter room to the red viscus preparation room (Figure 1), as described before. Insufficient cleaning of these surfaces both at the end of the slaughter day and throughout the workday contributed to the increased contamination of the organs. Additionally, we find it concerning that the viscera, upon exiting this conduit, pile up in a reservoir in the preparation room, remaining there until they are marked, washed, and transferred in carts to the refrigeration room. These facts compromise what is considered one of the primary benefits of vertical slaughterhouses: the achievement of meat with enhanced hygiene attributes resulting from a more distinct division between the clean and contaminated areas within the abattoir.

In summary, on the liver surface, an increase of nearly two logarithmic cycles (from 1.58 to 3.09 log CFU/cm^2^) for TMB and one logarithmic cycle (from 0.84 to 1.54 log CFU/cm^2^) for ENT between Point 1 and samples collected in the 3 °C refrigeration chamber (Point 3) was observed. Similarly, in the tongue samples, there was an increase of almost two logarithmic cycles for both TMB and ENT (from 1.63 to 3.19 log CFU/cm^2^ and from 0.38 to 2.20 log CFU/cm^2^ respectively). The counts of *S. aureus* remained stagnant throughout the viscus preparation process, which highlights its main association with handling during post-mortem inspection procedures (Point 1) and not with contact with contaminated surfaces.

## 4. Conclusions

With the exception of a few samples showing elevated ENT counts, most microbial levels met the microbiological standards outlined in the “Hygiene Criteria for Processes” for bovine carcasses under the Regulation EC 1441/2007 amendment of Regulation EC 2073/2005 [51]. However, the study results highlight significant concerns due to the high prevalence of pathogenic microorganisms, including presumptive *E. coli* O157, *S. aureus*, and *Salmonella*. This underscores the fact that the presence of pathogens is not necessarily correlated with high levels of spoilage bacteria. In general, the number of samples contaminated with pathogenic microorganisms and higher counts of spoilage microorganisms increase throughout the handling and preparation stages of offal, as well as on days with higher slaughter volumes. The results obtained reveal the need to implement measures in the slaughterhouse to avoid offal contamination.

This work prompted an improvement of the slaughterhouse HACCP plan, focusing on improving production and hygiene practices throughout the offal production process, particularly on improving the hygiene and disinfection plan of the viscus transport pipelines from the slaughter line to the preparation room.

Furthermore, it is essential to continue efforts to raise consumer awareness regarding the potential of offal as a viable and safe alternative source of animal protein. Ensuring the production of safe products for consumption is crucial to fostering trust and promoting offal consumption, which could significantly contribute to improving sustainability indicators in animal production.

## Figures and Tables

**Table 1:** Prevalence of presumptive *E. coli* O157, *Salmonella*, and *S. aureus* in liver and tongue samples.

Offal	Variables		N	Positive (%)		
				*E. coli* O157	*Salmonella*	*S. aureus*
Liver	Slaughter Volume	H	36	21.87%	0%	25%
		L	36	15.63%	2.77%	13.88%
	Harvest Point	1	24	12.5%	0%	12.5%
		2	24	20.83%	4.16%	29.17%
		3	24	16.66%	0%	16.66%
Tongue	Slaughter Volume	H	36	27.77%	0%	36.11%
		L	36	16.67%	11.11%	25%
	Harvest Point	1	24	25%	0%	8.33%
		2	24	20.83%	16.66%	29.16%
		3	24	20.83%	0%	54.16%

High and low slaughter volume (H and L). Sampling points: immediately after post-mortem inspection (Point 1); after the arrival of the viscera in the preparation room (Point 2); and in the refrigeration chamber at 3 °C (Point 3).

**Table 2:** Average counts of mesophilic microorganisms *Enterobacteriaceae*, generic *E. coli*, presumptive *E. coli* O157, and *S. aureus* (mean ± SD) from liver and tongue surface swabs. Average levels.

Offal	N	TMB		ENT		*E. coli*		*E. Coli* 0157		*S. aureus*	
		$\overset{-}{X}$	σ	$\overset{-}{X}$	σ	$\overset{-}{X}$	σ	$\overset{-}{X}$	σ	$\overset{-}{X}$	σ
Liver	72	2.49	0.94	1.36	1.04	0.30	0.62	0.26	0.57	0.47	1.94
Tongue	72	2.64	1.05	1.65	1.24	0.50	0.77	0.40	0.71	0.95	2.7
*p*-value		NS		NS		NS		NS		NS	

**Table 3:** (**a**). Average values of mesophilic *Enterobacteriaceae*, *E. coli*, *E. coli* O157, and *S. aureus* counts (Mean ± SD) of surface swabs of liver as a function of slaughter volume. (**b**). Average values of mesophilic *Enterobacteriaceae*, *E. coli*, *E. coli* O157, and *S. aureus* counts (Mean ± SD) of surface swabs of the tongue as a function of slaughter volume.

**(a)**											
**Slaughter** **Volume**	**N**	**TMB**		**ENT**		***E. coli***		***E. coli* O157**		***S. aureus***	
		** $\overset{-}{X}$ **	**σ**	** $\overset{-}{X}$ **	**σ**	** $\overset{-}{X}$ **	**σ**	** $\overset{-}{X}$ **	**σ**	** $\overset{-}{X}$ **	**σ**
H	36	2.75 ^a^	0.95	1.29	1.13	0.59 ^a^	0.77	0.44 ^a^	0.70	0.47	1.95
L	36	2.21 ^b^	0.84	1.44	0.97	0.00 ^b^	0.00	0.07 ^b^	0.32	0.47	1.96
*p*-value		**		NS		***		**		NS	
**(b)**											
**Slaughter** **Volume**	N	**TMB**		**ENT**		***E. coli***		***E. coli*** **O157**		***S. aureus***	
		** $\overset{-}{X}$ **	**σ**	** $\overset{-}{X}$ **	**σ**	** $\overset{-}{X}$ **	**σ**	** $\overset{-}{X}$ **	**σ**	** $\overset{-}{X}$ **	**σ**
H	36	3.02 ^a^	0.93	1.78	1.35	0.91 ^a^	0.89	0.61 ^a^	0.88	0.47	1.95
L	36	2.26 ^b^	1.03	1.52	1.12	0.00 ^b^	0.00	0.09 ^b^	0.32	1.43	3.24
*p*-value		**		NS		***		**		NS	

High slaughter (H), low slaughter (L); N—number of samples analysed; total mesophilic aerobic bacteria (TMB); *Enterobacteriaceae* (ENT); *p* < 0.01, very significant, (**), *p* < 0.001, highly significant (***), NS, non-significant. Different superscript letters (^a,b^) indicate statistical differences.

**Table 4:** (**a**) Average values of mesophilic *Enterobacteriaceae*, generic *E. coli*, presumptive *E. coli* O157, and *S. aureus* counts (mean ± SD) of surface swabs of the liver as a function of the sampling points: immediately after post-mortem inspection (Point 1); after the arrival of the viscera in the preparation room (Point 2); and in the refrigeration chamber at 3 °C (Point 3). (**b**) Average values of mesophilic *Enterobacteriaceae*, generic *E. coli*, presumptive *E. coli* O157, and *S. aureus* counts (mean ± SD) of surface swabs of the tongue as a function of the sampling points: immediately after post-mortem inspection (Point 1); after the arrival of the viscera in the preparation room (Point 2); and in the refrigeration chamber at 3 °C (Point 3).

**(a)**											
**Sampling** **Point**	**N**	**TMB**		**ENT**		***E. coli***		***E. coli*** **O157**		***S. aureus***	
		** $\overset{-}{X}$ **	**σ**	** $\overset{-}{X}$ **	**σ**	** $\overset{-}{X}$ **	**σ**	** $\overset{-}{X}$ **	**σ**	** $\overset{-}{X}$ **	**σ**
1	24	1.63 ^a^	1.00	0.38 ^a^	0.65	0.12 ^a^	0.34	0.00 ^a^	0.00	1.11	3.00
2	24	3.10 ^b^	0.79	2.37 ^b^	0.82	0.67 ^b^	0.75	0.76 ^b^	0.97	0.67	2.27
3	24	3.19 ^b^	0.39	2.20 ^b^	1.03	0.57 ^ab^	0.99	0.30 ^a^	0.55	1.06	2.86
*p*-value		***		***		*		***		NS	
**(b)**											
**Sampling** **Point**	**N**	**TMB**		**ENT**		***E. coli***		***E. coli*** **O157**		***S. aureus***	
		** $\overset{-}{X}$ **	**σ**	** $\overset{-}{X}$ **	**σ**	** $\overset{-}{X}$ **	**σ**	** $\overset{-}{X}$ **	**σ**	** $\overset{-}{X}$ **	**σ**
1	24	1.59 ^a^	0.79	0.84 ^a^	0.83	0.13	0.30	0.00 ^a^	0.00	0.00	0.00
2	24	2.78 ^b^	0.62	1.7 ^b^	0.94	0.23	0.51	0.24 ^ab^	0.50	0.66	2.23
3	24	3.09 ^b^	0.63	1.54 ^b^	1.17	0.53	0.85	0.53 ^b^	0.77	0.74	2.51
*p*-value		***		**		NS		**		NS	

N—number of samples analysed; total mesophilic aerobic bacteria (TMB); *Enterobacteriaceae* (ENT)*. p* < 0.05, significant, (*); *p* <0.01, very significant, (**), *p* < 0.001, highly significant (***), NS, non-significant. Different superscript letters (^a,b^) indicate statistical differences.

## Data Availability

All data are provided in the Supplementary Materials.

## Supplementary Materials

The following supporting information can be downloaded at: https://www.mdpi.com/article/10.3390/vetsci12020153/s1. Section S1: Chapman formulation; Section S2: *Enterobacteriaceae* counts (log CFU/cm^2^) at the different sampling points; Section S3: Microbial correlations between the tested microbial groups.
