# Supplementary material for: Sustainable Practices and Microbial Quality of Cattle Offal in Slaughterhouses
Source: Vet Sci. 2025 Feb 11;12(2):153. doi: 10.3390/vetsci12020153 (PMC11861329; doi:10.3390/vetsci12020153)
Supplement: Supplementary file 1 [file vetsci-12-00153-s001.zip › vetsci-3361110-supplementary.pdf]

Section S1

Formulation of Chapman broth medium:

- **Distilled water—200 mL**
- **Tryptone—2 g**
- **Proteose—2 g**
- **Meat extract—2.4 g**
- **Sodium chloride—30 g**
- **Agar—0.2 g**
- **Lactose—3 g**

Check the pH to ensure it is 7.4. Dispense 10 mL of the Chapman medium into 20 mm-diameter tubes, autoclave at 121 °C for 15 min.

Section S2 - *Enterobacteriaceae* counts (log CFU/cm²) at the different sampling points.

Sampling Point	Offal	*Enterobacteriaceae* (log UFC/cm²)
1	Liver	0.00
2	Liver	0.70
3	Liver	1.43
1	Tongue	1.00
2	Tongue	2.57
3	Tongue	1.00
1	Liver	1.95
2	Liver	1.40
3	Liver	0.00
1	Tongue	0.00
2	Tongue	2.23
3	Tongue	0.70
1	Liver	0.00
2	Liver	2.67
3	Liver	0.00
1	Tongue	0.00
2	Tongue	2.73
3	Tongue	1.00
1	Liver	0.00
2	Liver	1.00
3	Liver	2.04
1	Tongue	0.00
2	Tongue	3.09
3	Tongue	3.11
1	Liver	0.00
2	Liver	0.00
3	Liver	0.00
1	Tongue	0.00
2	Tongue	2.96
3	Tongue	1.65
1	Liver	2.59
2	Liver	1.60
3	Liver	1.18
1	Tongue	1.63
2	Tongue	1.65
3	Tongue	1.30
1	Liver	0.00
2	Liver	1.18
3	Liver	0.00
1	Tongue	0.00
2	Tongue	2.51

3	Tongue	2.64
1	Liver	0.00
2	Liver	0.00
3	Liver	1.48
1	Tongue	0.00
2	Tongue	0.00
3	Tongue	1.95
1	Liver	1.18
2	Liver	1.81
3	Liver	3.22
1	Tongue	1.95
2	Tongue	3.31
3	Tongue	3.52
1	Liver	1.48
2	Liver	3.05
3	Liver	2.58
1	Tongue	0.70
2	Tongue	3.26
3	Tongue	3.41
1	Liver	1.00
2	Liver	2.18
3	Liver	3.44
1	Tongue	0.00
2	Tongue	3.20
3	Tongue	3.99
1	Liver	1.78
2	Liver	2.07
3	Liver	3.30
1	Tongue	0.00
2	Tongue	3.11
3	Tongue	3.74
1	Liver	1.18
2	Liver	1.65
3	Liver	2.38
1	Tongue	0.00
2	Tongue	3.24
3	Tongue	2.47
1	Liver	0.00
2	Liver	2.60
3	Liver	2.38
1	Tongue	0.00
2	Tongue	2.85
3	Tongue	2.61
1	Liver	0.70
2	Liver	2.74

3	Liver	2.49
1	Tongue	0.00
2	Tongue	3.02
3	Tongue	2.51
1	Liver	0.00
2	Liver	2.85
3	Liver	0.00
1	Tongue	1.48
2	Tongue	1.89
3	Tongue	2.75
1	Liver	1.90
2	Liver	1.56
3	Liver	2.07
1	Tongue	0.00
2	Tongue	1.40
3	Tongue	1.64
1	Liver	0.00
2	Liver	2.41
3	Liver	1.98
1	Tongue	0.00
2	Tongue	1.54
3	Tongue	2.67
1	Liver	1.40
2	Liver	2.33
3	Liver	0.00
1	Tongue	0.00
2	Tongue	1.70
3	Tongue	1.00
1	Liver	1.81
2	Liver	0.00
3	Liver	1.40
1	Tongue	0.70
2	Tongue	1.78
3	Tongue	2.71
1	Liver	1.00
2	Liver	0.70
3	Liver	0.00
1	Tongue	0.00
2	Tongue	1.30
3	Tongue	2.69
1	Liver	0.00
2	Liver	1.44
3	Liver	1.40
1	Tongue	1.54
2	Tongue	2.18

3	Tongue	1.65
1	Liver	1.60
2	Liver	2.80
3	Liver	1.60
1	Tongue	0.00
2	Tongue	2.57
3	Tongue	2.15
1	Liver	0.70
2	Liver	1.99
3	Liver	2.65
1	Tongue	0.00
2	Tongue	2.79
3	Tongue	0.00

Point 1 - immediately after post-mortem inspection; Point 2 - after the arrival of the viscera in the preparation room; Point 3 - refrigeration chamber at 3 °C.

Section S3 - Microbial correlations between the tested microbial groups.

	**Mesophiles**	***Enterobacteriaceae***	***E. coli***	***E. coli* O157**	***S. aureus***
**Mesophiles**	----	0.000	0.000	0.000	0.515
Enterobacteriaceae	0.000	----	0.000	0.000	0.574
Escherichia coli	0.000	0.000	----	0.000	0.667
***E. coli* O157**	0.000	0.000	0.000	----	0.467
S. aureus	0.515	0.574	0.667	0.467	----

$p > 0.05$ indicates no correlation between the respective microbial groups.
